# Assembly processes of rhizosphere and phyllosphere bacterial communities in constructed wetlands created via transformation of rice paddies

**DOI:** 10.3389/fmicb.2024.1337435

**Published:** 2024-02-20

**Authors:** Nan Deng, Caixia Liu, Yuxin Tian, Qingan Song, Yandong Niu, Fengfeng Ma

**Affiliations:** ^1^Hunan Academy of Forestry, Changsha, Hunan, China; ^2^Hunan Cili Forest Ecosystem State Research Station, Cili, Changsha, Hunan, China; ^3^Dongting Lake National Positioning Observation and Research Station of Wetland Ecosystem of Hunan Province, Yueyang, China; ^4^International Technological Cooperation Base for Ecosystem Management and Sustainable Utilization of Water Resources in Dongting Lake Basin, Changsha, China

**Keywords:** constructed wetlands, rhizosphere bacterial community, phyllosphere bacterial community, degraded wetlands, community structure

## Abstract

Constructed wetlands are an efficient and cost-effective method of restoring degraded wetlands, in which the microorganisms present make a significant contribution to the ecosystem. In this study, we comprehensively investigated the patterns of diversity and assembly processes of 7 types of constructed wetlands at the rhizosphere and phyllosphere levels. The results showed that the rhizosphere communities of the constructed wetlands exhibited a more balanced structure than that of paddy fields, and 5 types of constructed wetland demonstrated higher potential diversity than that of paddy fields. However, the opposite trend was observed for the phyllosphere communities. Analysis of mean nearest taxon difference indicated that both deterministic and stochastic processes affected the establishment of the rhizosphere and phyllosphere communities, and stochastic processes may have had a larger effect. An iCAMP model showed that dispersal limitation was the most important factor (67% relative contribution) in the rhizosphere community, while drift was the most important (47% relative contribution) in the phyllosphere community. Mantel tests suggested that sucrase, average height, top height, total biomass, belowground biomass, maximum water-holding capacity, and capillary porosity were significantly correlated with processes in the rhizosphere community, whereas factors such as the deterministic process, average height, top height, and SOC were significantly correlated with deterministic processes in the phyllosphere community. Our results can assist in the evaluation of artificial restorations, and can provide understanding of the ecological processes of microbial communities, as well as new insights into the manipulation of microorganisms in polluted wetland ecosystems.

## 1 Introduction

As an indispensable type of ecosystem and landscape on Earth, wetlands are generated by the interdependence of water and land; this provides numerous ecosystem benefits, including habitats for flora and fauna, water quality enhancement, and carbon sequestration (Hu et al., 2017; Skinner, 2022). Wetlands are of particular relevance in reversing the eutrophication of surface water bodies, which is primarily caused by an influx of increased loads of phosphorus (P) due to changes in land use (Skinner, 2022). Driven by global climate change and human activities, wetlands presently face important issues, such as significant area shrinkage, gradual degradation, and even loss of their ecological functions (Chatterjee et al., 2015; Yuan et al., 2015). Due to the influence of human activities, such as changes in land use, the productivity and ecological functions of wetlands have been gradually degraded (Jiang et al., 2015). Constructed wetlands have been proven to be an efficient solution for wetland restoration: under this approach, a simulated ecosystem for the removal of pollutants is purposefully created (Guo et al., 2021; Li H. et al., 2021; Zhao et al., 2022). The removal of pollutants depends on complex interactions between plants and microorganisms (Bai et al., 2023). Microorganisms can convert organic matter into constituent substances and energy, thereby playing a core role in adsorbing and degrading pollutants (Kulikova and Perminova, 2021).

Recent studies of constructed wetlands have increasingly emphasized their potential in terms of pollutant removal and plant–soil/microorganism interactions (Wu et al., 2019; Nyer et al., 2022; Zhao et al., 2022). However, only a small number of studies have focused on the mechanisms of construction and patterns in diversity of the underlying microorganisms. Understanding the factors driving microbial distribution and diversity is a core area of research in microbial ecology (Martiny et al., 2006). Various fundamental patterns in biodiversity have been observed, such as latitudinal/altitude-based patterns (Alexander et al., 2011; Qian and Ricklefs, 2011), species–area relationships (SARs) (Liang et al., 2015), and species abundance distributions (SADs) (Matthews and Whittaker, 2014). However, the mechanisms underlying community construction and the factors that impact it remain controversial. The assembly of a microbial community is usually explained by deterministic processes and stochastic processes (Jiao and Lu, 2020). The notion of deterministic processes (based on traditional niche-based theory) rests on the hypothesis that community structures are governed by deterministic factors, such as species traits, interspecies interactions, and environmental conditions, while the notion of stochastic processes (under the neutral theory) rests on the view that community structures are governed by random factors, such as birth, death, colonization, and extinction (Chase and Myers, 2011). Studies of coastal wetlands indicate that inundation gradients and host plants determine patterns in the rhizosphere community, and homogeneous selection and dispersal limitation are the dominant factors in different groups (Gao et al., 2020). A study of wetland water microorganisms has also indicated that the assembly of different taxa is mainly governed by deterministic or stochastic processes (Yang et al., 2022). Additionally, most research on microbial community construction focuses on the rhizosphere community, while the phyllosphere community has been frequently overlooked, despite also playing an important role in the wetland ecosystem (Bashir et al., 2022).

In the present study, the study area was a constructed wetland established by returning farmland to wetland, with multiple disposition patterns. Deciphering and understanding the co-occurrence of the bacterial communities and details of their assembly can help us evaluate the effectiveness of artificial restoration and develop an understanding of the ecological processes involved, and may provide new insights into the manipulation of microorganism and ecosystem functions in wetland ecosystems. The main objectives of the present study were: (i) to explore the patterns of diversity in rhizosphere and phyllosphere bacteria in 7 constructed wetlands and paddy fields, and examine the differences between these communities; and (ii) to discover the process of assembly of the rhizosphere and phyllosphere communities of these 7 constructed wetlands and identify the dominant factor in community construction.

## 2 Materials and methods

### 2.1 Study area and sample collection

**Figure 1:**
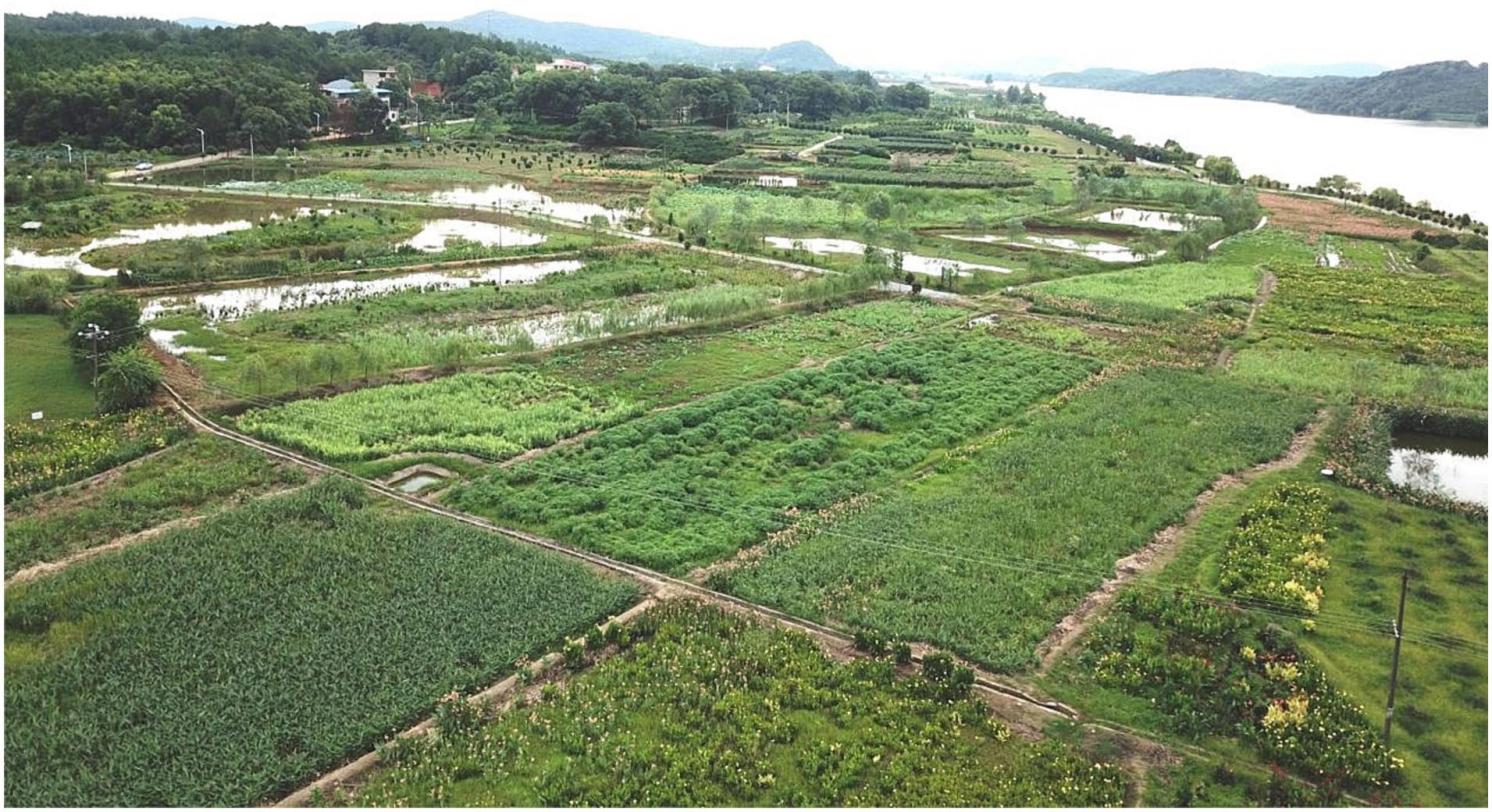
Sample plot layout.

The study area was located in an area designated for demonstration of returning farmland to wetland, Xiangjia Village, Yongzhou City, Hunan Province, China. This area belonged to the river wetlands in the middle reaches of the Xiangjiang River Basin (geographic coordinates of the center point: 111°45′58.0824″E, 26°34′36.6204”N). The land type was agricultural land that was densely populated. Wetland pollution is a relatively serious issue due to the perennial cultivation of rice and domestic sewage produced by surrounding residents. A series of measures were taken to recover these wetlands and build different types of constructed wetlands. Among these constructed wetlands, the measures of planting wetland plants have achieved a good ecological effect. Common wetland plants were experimentally combined, and the combinations associated with better growth and significant management effects were preserved. Plots representing each of the 7 constructed wetlands with different plant compositions were selected (Table 1; Figure 1). The area of each plot was approximately 800 m^2^, and plots were separated from one another by banks (40–50 cm). All the plots were constructed 3 years ago. Additionally, a plot representing paddy fields was also selected for comparison.

**Table 1:** Types of constructed wetlands with plant composition.

Type ID	Plant composition	Cover contribution (%)
1	*Canna indica L.*	90
2	*Cyperus alternifolius L.*	95
3	*Typha orientalis Presl*	95
4	*Thalia dealbata Fraser*	70
5	*Thalia dealbata* *Canna indica* *Typha orientalis*	30 30 30
6	*Cyperus alternifolius* *Typha orientalis*	50 40
7	*Arundo donax L.*	95
8	Rice	70

Within each plot, 3 small plots (1m^2^) were selected at intervals of more than 10 m; within these, measures of the growing status of plants were taken, such as height and coverage, and then all plants in the small plots were removed to collect samples. To obtain the rhizosphere soil, the topsoil (0–5 cm) was removed and the entire root system was dug out (20–40 cm), the plants were gently shaken, and the tightly bonded soil that remained attached to the root surface was then collected. All collected samples were immediately shipped to the laboratory in a 4°C environment. The soil sample (filtered using 2-mm mesh) was then divided into two parts: one part was stored at 4°C for physicochemical analysis, and the other was used for DNA extraction. To obtain the phyllosphere samples, healthy leaves were sampled from around the plants and immersed in 1 × PBS buffer (Coolaber, China) at 4°C, then shaken on a shaking table for 30 min (200 r/min); the oscillating solution was filtered using a 0.2-μm filter membrane. The entire experiment was conducted from August to September, which is the peak period of plant growth.

### 2.2 DNA extraction, sequencing, and bioinformatics analyses

Total microbial DNA was extracted using the FastDNA Spin Kit (MP Biomedicals, Santa Ana, CA), according to the manufacturer’s instructions. Bacterial 16S rRNA genes were amplified using the primers 515F (5′-GTGCCAGCMGCCGCGGTAA-3′) and 806R (5′-GACTACHVGGGTWTCTAAT-3′), tagged with unique barcodes for each sample. High-throughput sequencing was performed on an Illumina MiSeq platform (Illumina, Inc., San Diego, CA). Raw data sequences were processed and analyzed using QIIME2 (version: 2019) following the workflow described at https://qiime2.org (Bolyen et al., 2019). The UPARSE (v7.0.1) pipeline was used to select the operational taxonomic units (OTUs).

### 2.3 Physicochemical analyses

The plants were divided into aboveground and underground parts, which were assessed separately. Total biomass (kg/m^2^), aboveground biomass (kg/m^2^), and underground biomass (kg/m^2^) were calculated. A total of 15 soil and 4 leaf physicochemical properties were measured. Soil pH was determined by the potentiometric method; organic matter was determined by the potassium dichromate method; total salinity was determined by electrical conductance; total nitrogen was determined by the potassium dichromate–sulfuric acid digestion method; total phosphorus was determined by sulfuric acid–perchloric acid digestion and the Mo-Sb colorimetric method; total potassium was determined by NaOH-flame photometry; available nitrogen was determined by the Conway method; available phosphorus was determined by the NaHCO_3_ Mo-Sb colorimetric method; and available potassium was determined by the NH_4_OAc-flame photometry method. Catalase was determined by the permanganate titration method; sucrase was determined by the 3,5-dinitrosalicylic acid method; urease was determined by the indophenol blue colorimetry method; phosphatase was determined by the sodium diphenyl phosphate colorimetry method; protease was determined by the casein colorimetry method; and cadmium was determined by the atomic fluorescence spectrum method. A ring-knife was used to sample the soil for determination of permeability characteristics, non-capillary porosity, capillary porosity, total porosity, volume weight of soil, minimum water capacity, capillary water-holding capacity, and maximum water-holding capacity (Philip, 1957).

### 2.4 Analysis of bacterial community assembly processes

Rank abundance dominance (RAD) plots display the logarithm of species abundances against species rank order to analyze the types of abundance distributions present. In this study, 5 models were used, i.e., a broken stick model, a niche preemption model, a log-normal model, a Zipf model, and a Zipf–Mandelbrot model (Whittaker, 1965; Preston, 1984). The K-S test was used to test the models, and the Akaike Information Criterion (AIC) and Bayesian Information Criterion (BIC) were used to compare the models, with smaller AIC and BIC values taken to indicate a better fit. The Gambin model is an alternative approach that focuses on a single value that characterizes the shape of the SAD (Ugland et al., 2007). Gambin is a stochastic model that combines the γ-distribution with a binomial sampling method. A single free parameter (α) characterizes the distribution shape: low values indicate logseries-shaped curves and a higher proportion of rare species, whereas higher values indicate more lognormal-shaped curves (Matthews et al., 2019). We fitted the unimodal, bimodal, and trimodal versions of Gambin to the data, and then compared the three models using the BIC. The analyses of SAD and Gambin were conducted in R 4.1.3 using the packages ‘sad’ (Paulo et al., 2018) and ‘gambin’ (Thomas et al., 2021).

To characterize the phylogenetic composition of the community, we quantified the beta mean nearest taxon distance (betaMNTD) to quantify the turnover in phylogenetic composition over time; this is often coupled with randomization procedures (Stegen et al., 2012). To examine the relationship with β-diversity (Bray–Curtis distance), environmental distance and betaMNTD for the pairwise plots are presented. Mantel and partial Mantel analyses were carried out to examine the relationships between betaMNTD, environmental distance matrix, and diversity matrix. The matrices for β-diversity were divided into decomposed replacement, and richness differences, with the triplet values of replacement, richness difference, and similarity corresponding to a point in a triangular graph (Legendre, 2014). The above analysis was conducted using the R packages ‘picante’ (Steven et al., 2020), ‘vegan’ (Jari et al., 2019), and ‘ape’ (Paradis and Schliep, 2019).

To examine the soil and leaf bacterial community assembly processes, an iCAMP model was employed, using the ‘iCAMP’ package in R (Ning et al., 2020). The first step was phylogenetic binning, and five assembly processes were then examined via bin-based null model analysis. The five assembly processes were divided into deterministic processes (heterogeneous selection and homogeneous selection) and stochastic processes (dispersal limitation, homogenizing dispersal and drift, and others). Additionally, we computed the partial Mantel correlations between the phylogenetic bins and environmental variables, and assessed microbial niche breadth and overlap using the ‘spaa’ package (Zhang, 2016).

## 3 Results

### 3.1 Alpha diversity indices for rhizosphere and phyllosphere bacteria

**Figure 2:**
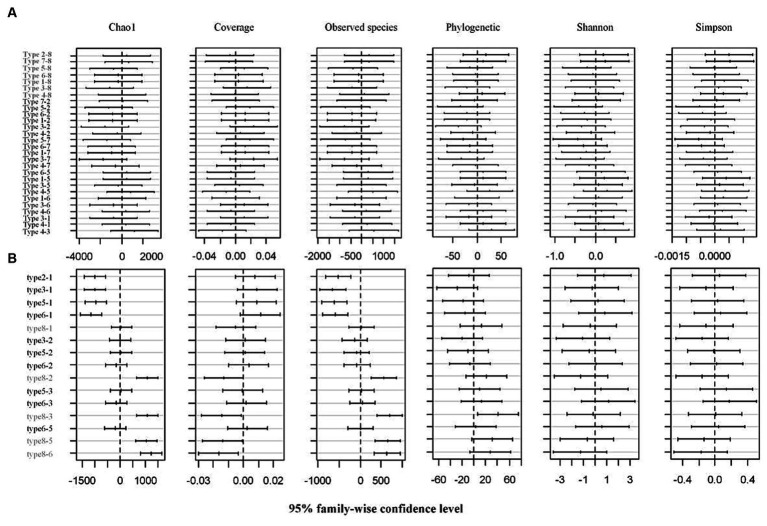
Multiple comparative analysis (Tukey HSD) of alpha diversity indices of **(A)** rhizosphere and **(B)** phyllosphere bacteria communities at 95% family-wise confidence level.

Soil physico-chemical properties and comparisons of different types of restored wetland are shown in Supplementary Figure S1. Total salinity content, organic matter, available phosphorus, and urease were at their highest in the rice plot. Conversely, cadmium and catalase content were lower in the rice plot than in other types (Supplementary Figure S1). After high-throughput sequencing, 16,278 and 3,220 OTUs were obtained from rhizosphere and phyllosphere types, respectively. The alpha diversity indices for the rhizosphere and phyllosphere are shown in Supplementary Table S1. The coverage exceeded 88% in the cases of 8 rhizosphere and 6 phyllosphere communities, indicating that the sequencing results were able to present a full reflection of the structure of the bacterial community. The other diversity indices showed no significant differences between the rhizosphere types (Figure 2A, *p* = 0.05). For the phyllosphere community, the Shannon and Simpson indices showed no significant differences between each type. However, it was found that the Chao1 and observed species indices were significantly higher for types 1 and 8 than for other types (Figure 2B, *p* = 0.05). In terms of the annotation results for rhizosphere communities, Proteobacteria, Chloroflexi, and Acidobacteria dominated the assigned class, and Anaeromyxobacter, Haliangium, Anaerolinea, and Geobacter dominated the assigned genus. For phyllosphere communities, Gammaproteobacteria, Alphaproteobacteria, and Actinobacteria dominated the assigned class, and Sphingomonas, Pseudomonas, and Methylobacterium dominated the assigned genus.

### 3.2 Results of SAD model fitting

**Figure 3:**
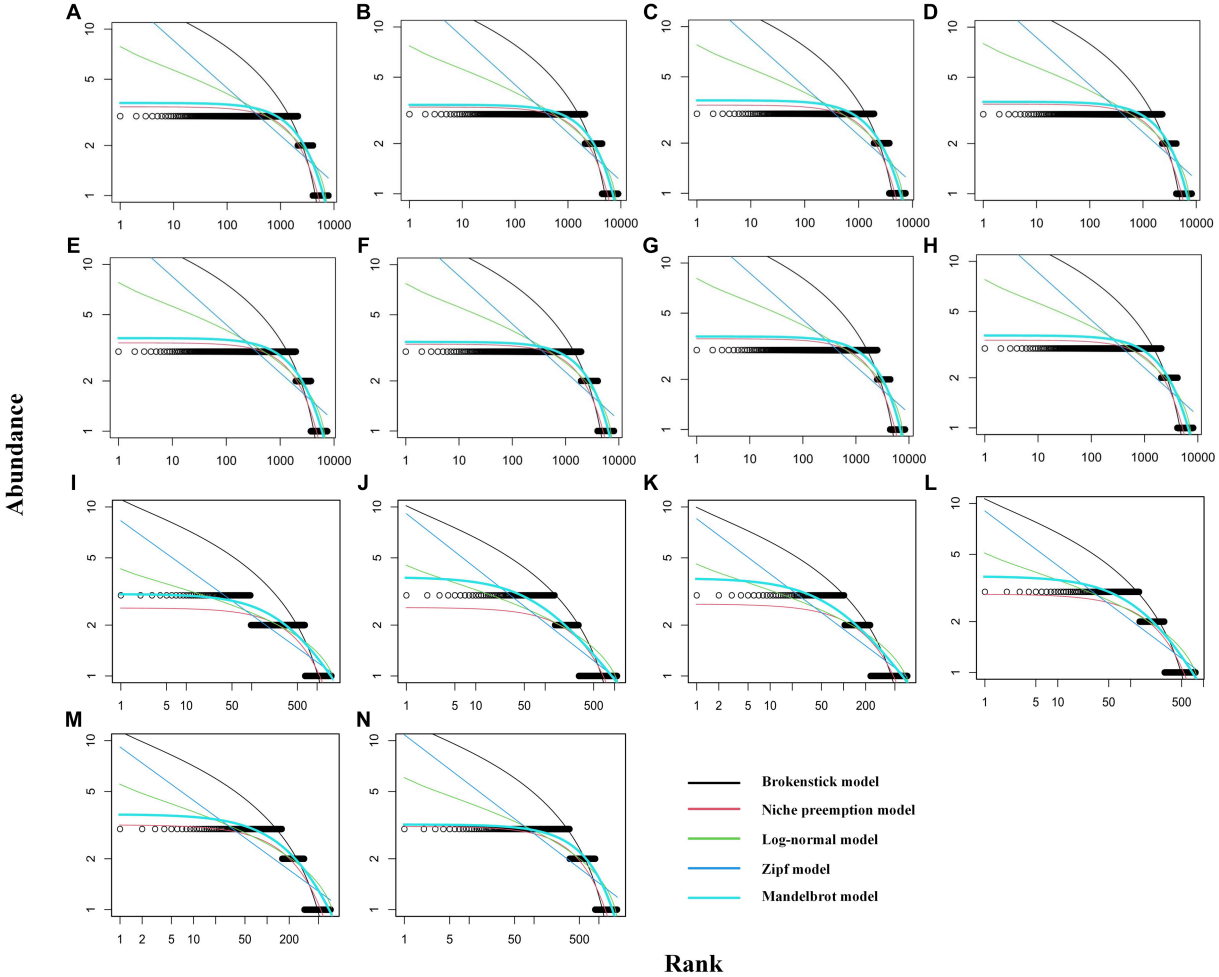
Curve-fitting plots for RAD. **(A–H)** Rhizosphere communities for plots of types 1–8; **(I–N)** phyllosphere communities for plots of types 1, 2, 3, 4, 5, 6, and 8.

The results of fitting the five SAD models are shown in Supplementary Tables S2, S3. The K-S test results indicated that all SAD models were accepted (*p* < 0.05), indicating that the SAD of these communities followed both logseries-and lognormal-like distributions. The curves fitted under the broken stick and Zipf–Mandelbrot models overlapped in the rhizosphere communities, indicating similar fitting effects (Figure 3). The AIC and BIC indicated that the Zipf–Mandelbrot model represented the best fit to all communities. The Zipf–Mandelbrot model was therefore selected for comparative analysis. The Zipf–Mandelbrot model fits the community structure via two parameters, beta and gamma. The gamma parameter takes low values in highly organized systems with complex interactions among species, while the beta parameter represents the potential diversity of the environment or niche diversification, taking higher values when the environment provides room for more alternatives (Frontier, 1985).

Based on the Zipf–Mandelbrot model of rhizosphere communities, the gamma parameter was the highest for plots of type 8, followed by type 3; it was lowest for type 7, indicating that the organizational structure of the rice community was less balanced. The beta parameter was the highest for type 7, followed by type 4; the parameter was lower for types 3, 5, and 8 than for other types, indicating that the communities of plots of types 3, 5, and 8 exhibited lower potential diversity than those of the other types (Table 2). Based on the Zipf–Mandelbrot model of phyllosphere communities, the gamma parameter was the highest for plots of type 2, followed by type 3; it was lowest for type 8, indicating that the organizational structure of the type 8 community was more balanced. The beta parameter was highest for type 8; the parameter was lower for types 2 and 3 than for other types, indicating that the communities of plots of types 2 and 3 exhibited lower potential diversity those of the other types (Table 2).

**Table 2:** Parameters fit under the Zipf–Mandelbrot and Gambin model.

Type of plot		Parameter of Zipf–Mandelbrot model		Gambin model
		Gamma	Beta	Alpha
Rhizosphere	Type 1	−7.1097	32,233	8.81
	**Type 2**	**−430.98**	**2.46E+06**	8.03
	**Type 3**	**−4.7321**	**19,095**	8.14
	Type 4	−64,180	3.34E+08	9.02
	Type 5	−4.7771	19,365	8.09
	**Type 6**	**−522.44**	**2.70E+06**	7.89
	Type 7	−788,600	4.21E+09	9.73
	Type 8	−4.6994	21,061	8.48
Phyllosphere	Type 1	−0.77515	396.09	3.9
	Type 2	−0.60245	87.55	1.74
	Type 3	−0.68941	86.89	2.22
	Type 5	−0.95494	193.11	3.42
	Type 6	−1.6454	469.34	5.24
	Type 8	−1.0831E+05	1.3655e+08	6.16

With respect to the Gambin model, unimodal, bimodal, and trimodal Gambin models were applied to fit SAD, and the unimodal Gambin model provided the best fit to all rhizosphere and phyllosphere communities. The higher alpha parameter indicated a strong diffusion restriction. In the results for the rhizosphere communities, the alpha parameter was highest for plots of type 7, followed by type 1, and this parameter was lowest in the case of 6. In the results for the phyllosphere communities, this parameter was highest for plots of type 8, and lowest in the case of type 2. The results indicated a weak diffusion limit in type 7 (the rhizosphere) and type 8 (the phyllosphere).

### 3.3 Bacterial community composition and distribution patterns

**Figure 4:**
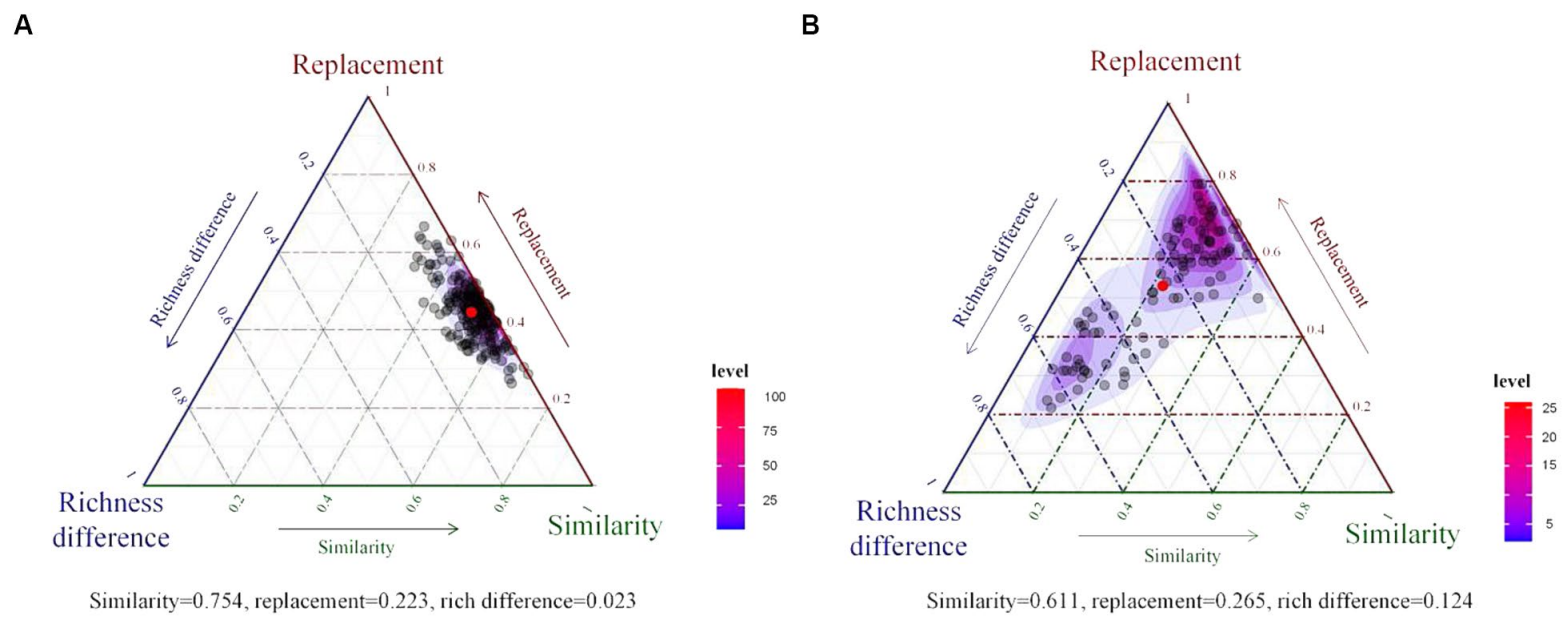
Ternary plots of beta diversity components for the rhizosphere **(A)** and phyllosphere **(B)** communities. The red dot represents the average value of all components.

Bacterial beta diversity was further partitioned into total replacement diversity and total richness difference diversity. The results indicated that dissimilarity in the bacterial community compositions of the rhizosphere (Figure 4A) and phyllosphere (Figure 4B) were dominated by species replacement processes. Replacement processes accounted for 22.3 and 26.5% of beta diversity in the rhizosphere and phyllosphere, respectively, and richness difference accounted for a low proportion.

**Figure 5:**
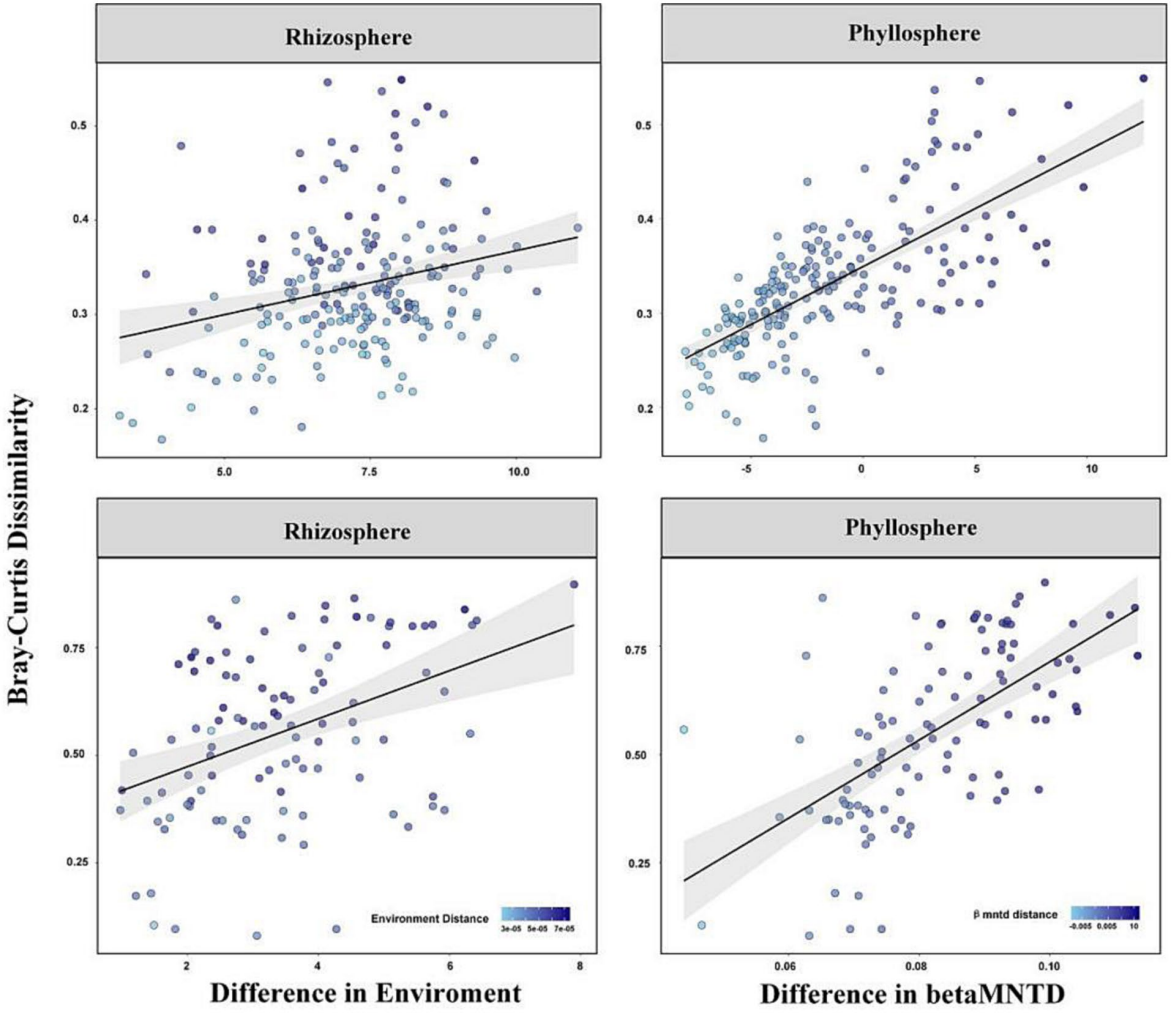
Variation in β-diversity with environmental distance and betaMNTD.

A linear regression between β-diversity and environmental distance/betaMNTD showed that the differences in species composition increased with increased environmental distance/betaMNTD among the rhizosphere and phyllosphere community (Figure 5). The fitted slope for betaMNTD was steeper than that for environmental distance in the case of both rhizosphere and phyllosphere community, indicating that stochastic processes dominated community construction. Among the rhizosphere and phyllosphere community, the Mantel test showed that β-diversity exhibited a significant positive correlation with both environment distance and betaMNTD. Partial Mantel test analysis indicated that β-diversity showed a significant positive correlation with environmental distance or betaMNTD when other factors were eliminated (*p* = 0.05, Table 3). The Mantel *r* value was higher for betaMNTD than for environmental distance in the case of both the rhizosphere and phyllosphere community. The results indicated that both deterministic processes and stochastic processes had effects on the establishment of the communities, and stochastic processes exerted a larger effect.

**Table 3:** Results of Mantel and partial Mantel tests between β-diversity and environmental distance/betaMNTD.

	M1	M2	M3	M4	Type
*r* statistic	0.26	0.36	0.71	0.73	Rhizosphere
Significance	0.013**	0.001***	0.001***	0.001***	
*r* statistic	0.41	0.38	0.61	0.60	Phyllosphere
Significance	0.015**	0.025*	0.001***	0.001***	

***, **, and * represent significance levels of 0.001, 0.01, and 0.05, respectively. M1–M4: environmental distance; environmental distance eliminating betaMNTD; betaMNTD; and betaMNTD eliminating environmental distance.

### 3.4 Bacterial community assembly processes

**Figure 6:**
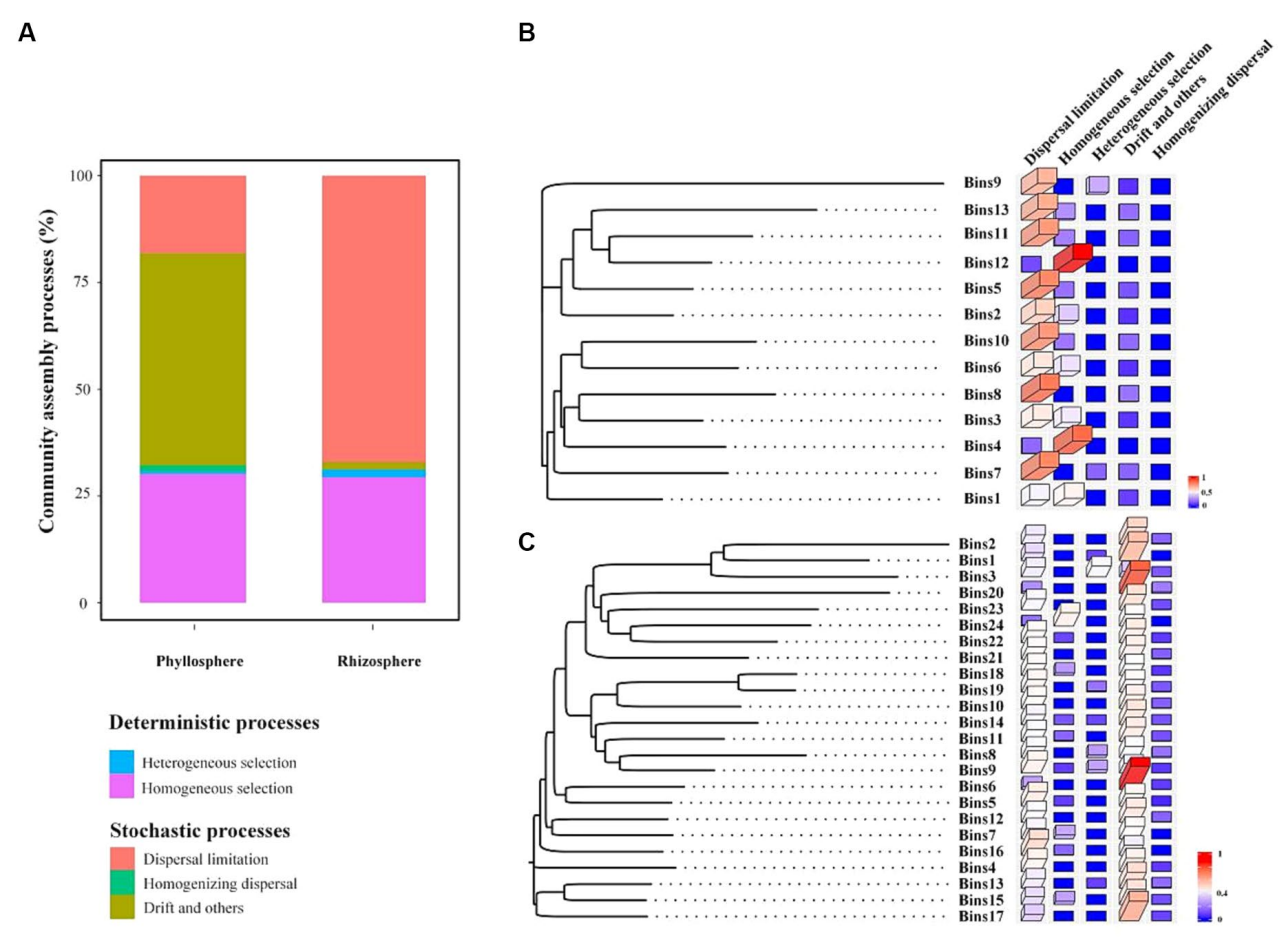
Relative contribution of each ecological process to the assembly of the whole community **(A)** and of each bin within the rhizosphere **(B)** and phyllosphere **(C)** communities.

The iCAMP analysis revealed that stochastic processes were the dominant processes in both the rhizosphere and phyllosphere communities (Figure 6A). Among the rhizosphere communities, dispersal limitation was the most important of the five processes, with a relative contribution of 67%. Among the rhizosphere communities, drift and others were the most important processes, with a relative contribution of 49.6%. Additionally, the OTUs of the rhizosphere community were divided into 13 phylogenetic bins (Figure 6B), and dispersal limitation was found to be the dominant process in most bins, with the exceptions of bins 1, 4, and 12 (where homogeneous selection was the dominant process). The OTUs of the phyllosphere community were divided into 24 phylogenetic bins (Figure 6C), and drift and others were the dominant processes in most bins, with the exceptions of bin 3 (heterogeneous selection), bin 24 (homogeneous selection), and bins 5, 9, 16, 18, and 19 (dispersal limitation).

**Figure 7:**
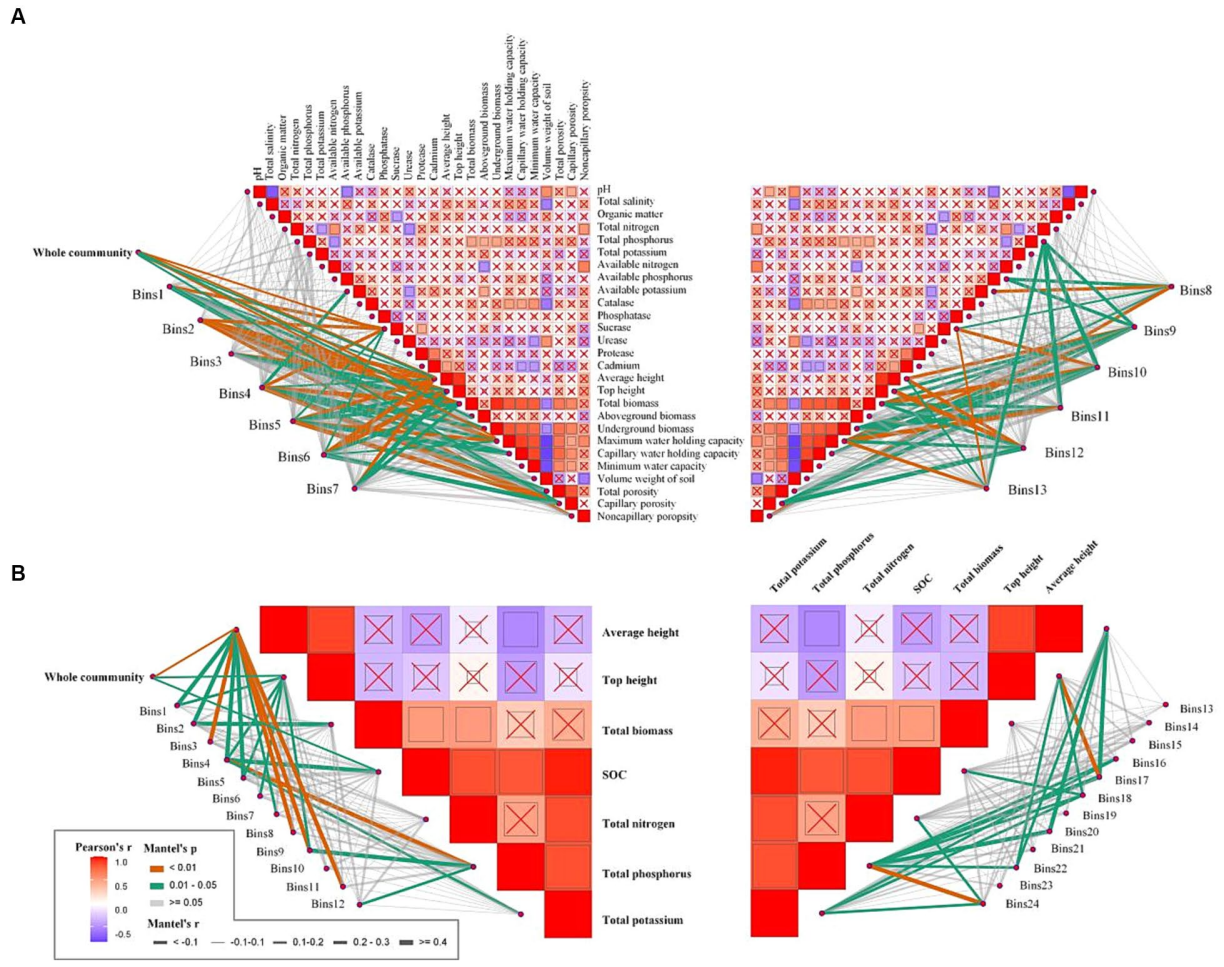
Environmental drivers of rhizosphere **(A)** and phyllosphere **(B)** community composition. Pairwise comparisons of environmental factors are shown, with a color gradient denoting Spearman’s correlation coefficients, and ‘x’ indicates no significant correlation. Community composition of bins was related to each environmental factor by Mantel tests (betaMNTD-corrected). Edge width corresponds to the Mantel’s r statistic, and edge color denotes the statistical significance based on 9,999 permutations.

To further investigate the environmental drivers of each bin in both communities, we correlated the betaMNTD-corrected dissimilarities of each bin composition with environmental factors. The results indicated that sucrase, average height, top height, total biomass, underground biomass, maximum water-holding capacity, and capillary porosity were significantly correlated with deterministic processes for the rhizosphere community (Figure 7A). In contrast, average height, top height, and SOC were significantly correlated with deterministic processes for the phyllosphere community (Figure 7B).

According to the iCAMP results for the rhizosphere community, homogeneous selection was the dominant process in bins 1, 4, and 12. Furthermore, sucrase, average height, total biomass, and capillary porosity were significantly correlated with bin 1; available potassium, sucrase, average height, total biomass, underground biomass, maximum water-holding capacity, total porosity, and capillary porosity were significantly correlated with bin 4; and top height, average height, total biomass, maximum water-holding capacity, and capillary porosity were significantly correlated with bin 12. Additionally, available phosphorus was significantly correlated with bins 8 and 9, and available potassium was significantly correlated with bins 4 and 8. The iCAMP results for phyllosphere community indicated that heterogeneous selection was the most important process in bin 3 except stochastic processes, and was significantly correlated with average height.

## 4 Discussion

Wetlands belong to the transitional ecological zone between terrestrial ecosystems and aquatic ecosystems, but they are affected by human activities such as the discharge of industrial and agricultural wastewater. China’s wetlands are facing ecological risks such as serious degradation of vegetation, rapid shrinkage of their area, and a drastic decline in ecosystem productivity. The government has taken a number of measures to improve the situation; the “Revert cultivated land to wetland” project has become an important measure for the protection of wetland resources and is being implemented gradually throughout the country. In this study, we carried out research on the microbial community building mechanisms underlying the most successful restoration model, which provides an important basis for evaluating the project of converting retired farmland to wetland. Different SAD models are often used to verify the different mechanisms governing species assembly (Matthews et al., 2017). SAD models are often used to detect disturbance and damage to the ecosystem and to explain resource allocation and interspecific associations among the species present (Hill and Hamer, 2002; Sugihara et al., 2003; Chase, 2005). These processes are not mutually exclusive; thus, the comparison of SAD models is essential to reveal the relative contributions to the SAD pattern (Volkov et al., 2003). A broken stick model is often used to represent small homogeneous communities with stable population, and a niche preemption model is used to describe both simple and complex communities (MacArthur, 1957). The suitability of a log-normal model indicates that the communities are dominated by random processes, and a Zipf–Mandelbrot model can provide support for hypotheses pertaining to underlying processes, linking the requirements of various species with probabilities of encountering optimal growth conditions in the environment (Barange and Campos, 1991). In this case, the model-fitting results indicated that the SAD followed more than one rule. According to the result of best-fitting model, the rhizosphere was more balanced in most constructed wetlands than in rice, and the former provided more natural resources for niche diversification. In contrast, the phyllosphere community showed the opposite trend. The rice soil in this area had undergone long-term eutrophication because of fertilization, creating an an extremely beneficial soil habitat for certain species. The fertilization promoted the growth of rice leaf and provided more resources to the phyllosphere community.

In this study, stochastic processes were identified as the dominant ecological processes in both the rhizosphere and phyllosphere communities, but the dominant factors differed in each case. Dispersal limitation was the most important factor (67% relative contribution) in the rhizosphere community, and drift and others were the most important (47% relative contribution) in the phyllosphere community. Ecological drift is a critical concept in community ecology (Zhou and Ning, 2017), and is particularly important in a small community with weak selection (Chase and Myers, 2011). Compared with the rhizosphere environment, the environment of a phyllosphere community is hostile, and involves temperature fluctuations, ultraviolet radiation, and so on (Bashir et al., 2022). The extreme and fast-changing environment causes more frequent extinction events, which is also evident in Figure 4B (the rich difference component accounted for a much higher proportion than in the case of the rhizosphere community). In addition, functional redundancy may be one of the causes of drift (Li Q. et al., 2021; Ren et al., 2022).

As a fundamental process in ecology, the dispersal process cannot be unambiguously treated as either deterministic or stochastic (Lowe and McPeek, 2014; Vellend et al., 2014). The dispersal processes of microbial communities are poorly understood because these communities are small in size and have high abundance, wide distributions, and short generation times (Nemergut et al., 2013; Evans et al., 2017). Whether microorganisms are dispersal-limited is still a controversial question, and previous studies suggest that microorganisms are not dispersal-limited (Finlay, 2002; Barbour et al., 2023). Recent studies have provided some evidence of dispersal limitation, such as the strong biogeographic patterns of microorganisms (Feng et al., 2019; Mony et al., 2020; Li Y. et al., 2021), and dispersal through small soil pores of a microorganism can be affected by its size or shape (Zhou and Ning, 2017). Both deterministic and stochastic factors may coexist in the dominant dispersal process in the rhizosphere community. On one hand, the plants in a constructed wetland form a diverse rhizosphere environment by altering the soil structure (Jacoby et al., 2021); on other hand, plant root exudates play a major role in determining the outcome of individual-and community-level chemical interactions (O'Banion et al., 2020), with some microbiome members being specifically recruited (Stopnisek and Shade, 2021), which can be inferred from the results of the Mantel test correlating β-diversity and environmental factors (Figure 7A).

**Figure 8:**
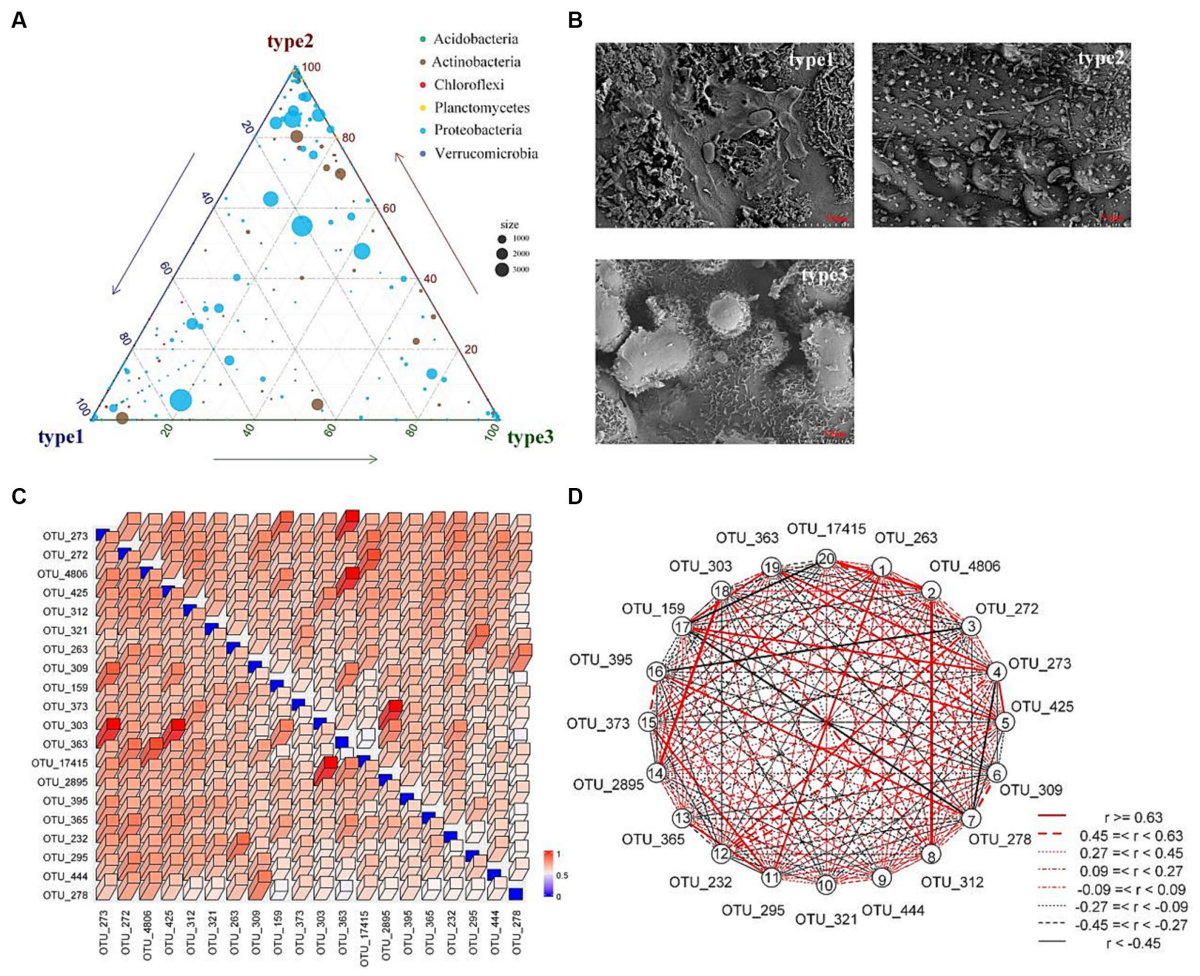
Community composition and niche overlap. **(A)** Triangle diagram of phyllosphere community species composition. **(B)** SEM images of particulate matter morphology on leaf surface. **(C)** Heatmap of Levin’s overlap calculated on the top 20 OTUs filtered from bins 4, 8, and 9 of the rhizosphere community. **(D)** Correlations between the top 20 OTUs.

In general, homogeneous selection dominates the assembly of prokaryotic communities (Wang et al., 2022). Soil physicochemical properties are the primary determinants of the root-associated construction of the bacterial community, followed by environmental factors, host genotype, and nutrient availability (Ren et al., 2020; Stopnisek and Shade, 2021). In this study, homogeneous selection was found to be the dominant factor in the deterministic ecological processes of both types of communities. The area studied was constructed from rice fields with highly homogenous soil physicochemistry and microclimate, which may be the main reason for homogeneous selection in both communities. According to the results of Mantel tests, the deterministic process of the rhizosphere community was significantly correlated with sucrase, plant growth characteristics (average height, top height, total biomass, and underground biomass), maximum water-holding capacity, and capillary porosity. Among these factors, sucrase is closely related to soil organic matter accumulation (Madejón et al., 2007), while maximum water-holding capacity and capillary porosity characterize aspects of the soil structure that are related to plant growth. The average height, top height, total biomass, and underground biomass reflect the differences in host plant genotype to some extent. Similarly to the rhizosphere community, the key factors in construction of the phyllosphere community were average height, top height, and SOC. SOC reflects the carbon source that is provided by the leaf, which is the sole carbon and energy source for bacteria (Abanda-Nkpwatt et al., 2006; Jaeger et al., 2018). The roles of average height and top height reflect aspects of the microclimate, such as solar radiation, wind, and humidity (Bashir et al., 2022), the impact of which is largely determined by species. Additionally, the complex epicuticular architecture also exerted an effect on the community composition (Figure 8A); specifically, type 3 showed a deep groove with scale-like waxy structure, type 2 was relatively smooth with many small protrusions, and type 1 had a dense sheet-like crystal structure (Figure 8B). Among the bins of the rhizosphere community, bins 4, 8, and 9 exhibited significant correlations with the available phosphorus and potassium, indicating that the microorganisms in these bins may be related to the removal of these pollutants. The top 20 OTUs among bins 4, 8, and 9 were filtered for assessment of the breath of the microbial niche and overlap. The results indicated that there was little difference between each OTU in terms of niche breadth, and the niches of these OTUs overlapped heavily (Figure 8C). Furthermore, many OTUs exhibited strong correlations with others (Figure 8D), indicating that these OTUs compete for the same resources (Finn et al., 2020) and their functions are redundant. Overall, the species of the host plant and nutrient availability played an important role in the constructions of rhizosphere and phyllosphere communities of the constructed wetlands.

The adaptability of plants to various environments does not stem solely from the plant genome itself. Rhizosphere microorganisms also have a positive impact on the host’s adaptation to stress, which is an effective extension of the plant genome (Hashem et al., 2019; Keswani et al., 2019). Host plants can mold the rhizosphere in favor of microbial communities that are beneficial to their own growth and metabolism through complex changes in the composition of rhizosphere secretions (Tkacz et al., 2015; Pii et al., 2016; Sánchez-Cañizares et al., 2017). Thus, inoculating healthy soil with “donor” microorganisms in small amounts can help to restore degraded ecosystems, which may represent a new idea for the restoration of degraded wetlands (Wubs et al., 2016). Examples of this strategy could include stimulation of functional groups of bacteria via management practices (van Agtmaal et al., 2015); addition of exogenous precursors for metabolic pathways that can modulate the production of nutritional, biocontrol, and anti-stress products by indigenous soil bacteria (Garbeva and Weisskopf, 2020); and customizing an effective rhizosphere bacterial community with desirable traits for specific purposes such as nitrogen and phosphorus solubilization though synthetic biology and genetical engineering (Haskett et al., 2022; Pantigoso et al., 2022). The types of restoration examined in this study can serve as an important reference point and lesson for the restoration of other polluted wetlands, and the findings can be extended to other areas in the Xiangjiang River Basin. Additionally, our study can assist in broadening the field’s understanding of microbial structures and the microbial species with potential for removal of pollutants; however, in-depth research on the evaluation and application of these species remains necessary.

## 5 Conclusion

This article has presented results on the patterns of diversity and community assembly observed in 7 types of constructed wetlands at the rhizosphere and phyllosphere level. We found that the paddy field exhibited a less balanced structure in the rhizosphere community and a more stable phyllosphere community, which was caused by eutrophication in this area. Both deterministic and stochastic processes affected the establishment of the rhizosphere and phyllosphere communities, but stochastic processes exerted a stronger effect. Among the stochastic processes at work, dispersal limitation was the most important in the rhizosphere community and drift was the most important in the phyllosphere community; this difference may be caused by the microclimate and the size of communities. Partial Mantel tests of the relationships between deterministic processes and environmental factors showed that the plant species played an important role in community construction. Our results can provide help in understanding the patterns of diversity and assembly mechanisms underlying both the rhizosphere and the phyllosphere communities, thereby providing a scientific basis for future manipulation of microorganisms in polluted wetland ecosystems.

## Data availability statement

The original contributions presented in the study are included in the article/Supplementary material, further inquiries can be directed to the corresponding author.

## Author contributions

ND: Writing – original draft. CL: Data curation, Investigation, Writing – original draft, Writing – review & editing. YT: Conceptualization, Writing – original draft, Writing – review & editing. QS: Software, Writing – original draft, Writing – review & editing. YN: Methodology, Writing – review & editing. FM: Funding acquisition, Resources, Writing – original draft.
