# Supplementary material for: Assembly processes of rhizosphere and phyllosphere bacterial communities in constructed wetlands created via transformation of rice paddies
Source: Front Microbiol. 2024 Feb 20;15:1337435. doi: 10.3389/fmicb.2024.1337435 (PMC10913029; doi:10.3389/fmicb.2024.1337435)
Supplement: Supplementary file 1 [file Table_1.docx]

**Supplementary table 1** Alpha diversity metrics of rhizosphere and phyllosphere bacteria community

| **Type** | | **Chao1** | **Coverage** | **Observed species** | **Phylogenetic** | **Shannon** | **Simpson** |
| --- | --- | --- | --- | --- | --- | --- | --- |
| rhizosphere | Type1 | 7830.92±512.26 | 0.8975±0.0073 | 4660.60±284.62 | 251.58±12.50 | 10.78±0.15 | 0.9986±0.0002 |
|  | Type2 | 8675.79±1025.26 | 0.8854±0.0136 | 5119.17±467.04 | 272.49±16.19 | 10.99±0.24 | 0.9987±0.0002 |
|  | Type3 | 7064.81±844.75 | 0.9084±0.0129 | 4342.23±404.05 | 233.89±20.17 | 10.64±0.15 | 0.9983±0.0001 |
|  | Type4 | 8203.22±71.81 | 0.8915±0.0004 | 4896.90±48.50 | 264.29±3.38 | 10.87±0.05 | 0.9985±0.0001 |
|  | Type5 | 7410.88±711.96 | 0.9040±0.0090 | 4364.50±317.46 | 238.34±10.61 | 10.58±0.17 | 0.9982±0.0002 |
|  | Type6 | 7831.03±462.87 | 0.8974±0.0070 | 4662.17±389.01 | 251.54±18.06 | 10.72±0.26 | 0.9983±0.0004 |
|  | Type7 | 8811.14±520.81 | 0.8852±0.0067 | 5106.77±269.69 | 267.14±12.60 | 11.02±0.13 | 0.9987±0.0001 |
|  | Type8 | 8203.77±556.36 | 0.8931±0.0071 | 4775.77±242.67 | 253.95±7.31 | 10.79±0.15 | 0.9982±0.0003 |
| phyllosphere | Type1 | 1676.81±147.74 | 0.99±0.01 | 1003.53±41.55 | 66.44±19.52 | 4.48±0.50 | 0.8748±0.0606 |
|  | Type2 | 653.61±225.80 | 0.99±0.01 | 476.23±158.02 | 57.73±13.32 | 5.25±0.44 | 0.9252±0.1234 |
|  | Type3 | 659.87±82.50 | 0.99±0.001 | 358.00±99.65 | 38.22±6.10 | 4.19±1.36 | 0.7643±0.2136 |
|  | Type5 | 690.09±34.36 | 0.99±0.001 | 399.60±29.98 | 48.03±1.41 | 4.72±0.02 | 0.9057±0.0015 |
|  | Type6 | 501.92±41.78 | 1.00±0.00 | 404.30±27.01 | 51.45±2.20 | 5.32±0.06 | 0.9442±0.0014 |
|  | Type8 | 1726.94±114.63 | 0.98±0.001 | 1043.90±95.57 | 78.70±6.18 | 4.04±0.68 | 0.7649±0.09146 |

**Supplementary table 2**. Fitting results of 5 SAD models among 8 rhizosphere bacteria community.

| **Type** | **Model** | **Constant parameter** | **Gamma parameter** | **Beta parameter** | **Deviation** | **Akaike Information Criterion** | **Bayesian Information Criterion** |
| --- | --- | --- | --- | --- | --- | --- | --- |
| Type1 | M1 | / | / | / | 5891.18 | 24848.97 | 24848.97 |
|  | M2 | 2.44E-4 | / | / | 843.03 | 19802.81 | 19809.78 |
|  | M3 | 0.494 | 0.409 | / | 726.4 | 19688.19 | 19702.13 |
|  | M4 | 1.18E-3 | -0.285 | / | 1347.56 | 20309.35 | 20323.29 |
|  | M5 | 2.94E+28 | -7.1106 | 32233 | 376.75 | 19340.54 | 19361.44 |
| Type2 | M1 | / | / | / | 6578.02 | 27770.33 | 27770.33 |
|  | M2 | 2.155E-4 | / | / | 933.36 | 22127.67 | 22134.76 |
|  | M3 | 0.469 | 0.408 | / | 751.34 | 21947.65 | 21961.82 |
|  | M4 | 1.11E-3 | -0.288 | / | 1365.75 | 22562.06 | 22576.23 |
|  | M5 | ∞ | -430.98 | 2.46E+06 | 411.46 | 21609.77 | 21631.03 |
| Type3 | M1 | / | / | / | 5509.37 | 23297.81 | 23297.81 |
|  | M2 | 2.60E-4 | / | / | 790.48 | 18580.92 | 18587.82 |
|  | M3 | 4.82E-01 | 0.411 | / | 680.57 | 18473.01 | 18486.82 |
|  | M4 | 1.26E-3 | -0.288 | / | 1240.33 | 19032.77 | 19046.58 |
|  | M5 | 5.00E+16 | -4.732 | 19095 | 355.22 | 18149.66 | 18170.38 |
| Type4 | M1 | / | / | / | 6164.34 | 25970.23 | 25970.23 |
|  | M2 | 2.35E-04 | / | / | 888.75 | 20696.64 | 20703.65 |
|  | M3 | 0.503 | 0.409 | / | 782.87 | 20592.75 | 20606.77 |
|  | M4 | 1.13E-3 | -0.284 | / | 1457.21 | 21267.1 | 21281.11 |
|  | M5 | ∞ | -64180 | 3.34E+08 | 406.31 | 20218.2 | 20239.22 |
| Type5 | M1 | / | / | / | 5533.67 | 23404.8 | 2.34E+04 |
|  | M2 | 2.56E-4 | / | / | 795.45 | 18668.58 | 18675.49 |
|  | M3 | 0.483 | 0.411 | / | 688.85 | 18563.98 | 18577.81 |
|  | M4 | 1.25E-3 | -0.288 | / | 1253.74 | 19128.87 | 19142.7 |
|  | M5 | 8.33E+16 | -4.777 | 19365 | 359.55 | 18236.68 | 18257.42 |
| Type6 | M1 | / | / | / | 5970.93 | 25241.19 | 25241.19 |
|  | M2 | 2.38E-4 | / | / | 850.82 | 20123.09 | 20130.08 |
|  | M3 | 0.47 | 0.409 | / | 700.77 | 19975.03 | 19989.01 |
|  | M4 | 1.19E-3 | -0.289 | / | 1268.23 | 20542.49 | 20556.48 |
|  | M5 | ∞ | -522.44 | 2.70E+06 | 381.95 | 19658.21 | 19679.18 |
| Type7 | M1 | / | / | / | 6396.55 | 26826.6 | 26826.6 |
|  | M2 | 2.29E-4 | / | / | 934.16 | 21366.22 | 21373.25 |
|  | M3 | 0.52 | 0.408 | / | 839.21 | 21273.27 | 21287.34 |
|  | M4 | 1.09E-3 | -0.281 | / | 1578.49 | 22012.54 | 22026.61 |
|  | M5 | ∞ | -7.89E+05 | 4.21E+09 | 430.85 | 20866.9 | 20888.01 |
| Type8 | M1 | / | / | / | 6082.02 | 25673.83 | 25673.83 |
|  | M2 | 2.35E-4 | / | / | 864.31 | 20458.11 | 20465.12 |
|  | M3 | 0.482 | 0.408 | / | 719.6 | 20315.4 | 20329.41 |
|  | M4 | 1.16E-3 | -0.287 | / | 1325.27 | 20921.08 | 20935.09 |
|  | M5 | 5.20E+16 | -4.699 | 21061 | 375.98 | 19973.78 | 19994.8 |

Note: M1-M5 represent the brokenstick, niche preemption model, log-normal Zipf and Zipf-Mandelbrot model

**Supplementary table 3**. Fitting results of 5 SAD models among 6 phyllosphere bacteria community.

| **Type** | **Model** | **Constant parameter** | **Gamma parameter** | **Beta parameter** | **Deviation** | **Akaike Information Criterion** | **Bayesian Information Criterion** |
| --- | --- | --- | --- | --- | --- | --- | --- |
| Type1 | M1 | / | / | / | 1313.92 | 5076.36 | 5076.36 |
|  | M2 | 1.05E-3 | / | / | 214.24 | 3978.67 | 3984.09 |
|  | M3 | 0.312 | 0.345 | / | 96.66 | 3863.10 | 3873.93 |
|  | M4 | 3.44E-3 | -0.279 | / | 115.37 | 3881.81 | 3892.64 |
|  | M5 | 0.131 | -0.775 | 396.09 | 67.12 | 3835.56 | 3851.81 |
| Type2 | M1 | / | / | / | 823.39 | 3237.76 | 3237.76 |
|  | M2 | 1.660E-3 | / | / | 164.93 | 2581.30 | 2586.29 |
|  | M3 | 0.265 | 0.387 | / | 96.88 | 2515.26 | 2525.24 |
|  | M4 | 5.967E-3 | -0.322 | / | 87.94 | 2506.32 | 2516.30 |
|  | M5 | 0.037 | -0.602 | 87.55 | 49.96 | 2470.33 | 2485.31 |
| Type3 | M1 | / | / | / | 547.90 | 2210.23 | 2210.23 |
|  | M2 | 2.48E-03 | / | / | 103.66 | 1767.99 | 1772.60 |
|  | M3 | 0.293 | 0.397 | / | 64.31 | 1730.64 | 1739.86 |
|  | M4 | 7.93E-3 | -0.327 | / | 64.59 | 1730.92 | 1740.14 |
|  | M5 | 0.077 | -0.689 | 86.89 | 33.58 | 1701.91 | 1715.74 |
| Type5 | M1 | / | / | / | 561.51 | 2345.02 | 2345.02 |
|  | M2 | 2.42E-03 | / | / | 94.80 | 1880.31 | 1884.97 |
|  | M3 | 0.349 | 0.410 | / | 66.54 | 1854.05 | 1863.36 |
|  | M4 | 7.53E-3 | -0.323 | / | 83.69 | 1871.20 | 1880.51 |
|  | M5 | 0.471 | -0.955 | 193.11 | 35.50 | 1825.02 | 1838.99 |
| Type6 | M1 | / | / | / | 526.81 | 2251.32 | 2251.32 |
|  | M2 | 2.61E-3 | / | / | 80.84 | 1807.35 | 1811.95 |
|  | M3 | 0.416 | 0.417 | / | 65.66 | 1794.17 | 1803.37 |
|  | M4 | 7.56E-3 | -0.316 | / | 100.24 | 1828.75 | 1837.95 |
|  | M5 | 75.21 | -1.645 | 469.34 | 35.20 | 1765.71 | 1779.50 |
| Type8 | M1 | / | / | / | 1395.57 | 5874.28 | 5874.28 |
|  | M2 | 9.89E-4 | / | / | 204.86 | 4685.58 | 4691.13 |
|  | M3 | 0.42 | 0.404 | / | 145.13 | 4627.84 | 4638.94 |
|  | M4 | 3.57E-3 | -0.301 | / | 234.62 | 4717.33 | 4728.44 |
|  | M5 | Inf | -1.08E+05 | 1.37E+08 | 92.18 | 4576.89 | 4593.54 |

Note: M1-M5 represent the brokenstick, niche preemption model, log-normal Zipf and Zipf-Mandelbrot model
