# Supplementary material for: Assembly processes of rhizosphere and phyllosphere bacterial communities in constructed wetlands created via transformation of rice paddies
Source: Front Microbiol. 2024 Feb 20;15:1337435. doi: 10.3389/fmicb.2024.1337435 (PMC10913029; doi:10.3389/fmicb.2024.1337435)
Supplement: Supplementary file 2 [file Image_1.pdf]

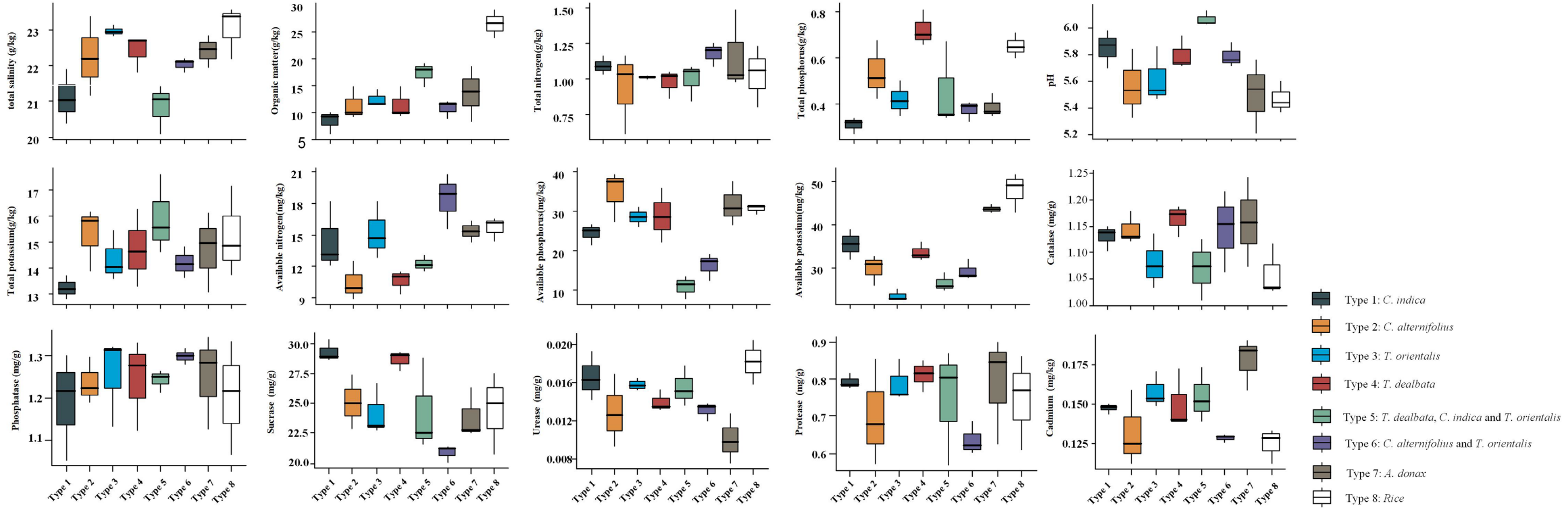

Figure S1 Physicochemical characteristics of constructed wetland and rice field rhizosphere soils. Boxplot represent the content of total salinity, organic matter, total nitrogen, total phosphorus, total potassium, available nitrogen, available phosphorus and available potassium, respectively. The color of boxplot represent different constructed wetland types and rice field.
